# Evaluation of target dose inhomogeneity in breast cancer treatment due to tissue elemental differences

**DOI:** 10.1186/s13014-018-1022-1

**Published:** 2018-05-15

**Authors:** A. Fogliata, F. De Rose, A. Stravato, G. Reggiori, S. Tomatis, M. Scorsetti, L. Cozzi

**Affiliations:** 1 Humanitas Research Hospital and Cancer Center, Radiotherapy and Radiosurgery Dept, Via Manzoni 56, 20089 Milan, Rozzano Italy; 2 Humanitas University, Biomedical Science Faculty, Milan, Rozzano Italy

**Keywords:** Breast tissue, Monte Carlo, Tissue assignment, Dose to medium

## Abstract

**Background:**

Monte Carlo simulations were run to estimate the dose variations generated by thedifference arising from the chemical composition of the tissues.

**Methods:**

CT datasets of five breast cancer patients were selected. Mammary gland was delineated as clinical target volume CTV, as well as CTV_lob and CTV_fat, being the lobular and fat fractions of the entire mammary gland. Patients were planned for volumetric modulated arc therapy technique, optimized in the Varian Eclipse treatment planning system. CT, structures and plans were imported in PRIMO, based on Monte Carlo code Penelope, to run three simulations: AdiMus, where the adipose and muscle tissues were automatically assigned to fat and lobular fractions of the breast; Adi and Mus, where adipose and muscle, respectively were assigned to the whole mammary gland. The specific tissue density was kept identical from the CT dataset. Differences in mean doses in the CTV_lob and CTV_fat structures were evaluated for the different tissue assignments. Differences generated by the tissue composition and estimated by Acuros dose calculations in Eclipse were also analysed.

**Results:**

From Monte Carlo simulations, the dose in the lobular fraction of the breast, when adipose tissue is assigned in place of muscle, is overestimated by 1.25 ± 0.45%; the dose in the fat fraction of the breast with muscle tissue assignment is underestimated by 1.14 ± 0.51%. Acuros showed an overestimation of 0.98 ± 0.06% and an underestimation of 0.21 ± 0.14% in the lobular and fat portions, respectively. Reason of this dissimilarity resides in the fact that the two calculations, Monte Carlo and Acuros, differently manage the range of CT numbers and the material assignments, having Acuros an overlapping range, where two tissues are both present in defined proportions.

**Conclusion:**

Although not clinically significant, the dose deposition difference in the lobular and connective fat fraction of the breast tissue lead to an improved knowledge of the possible dose distribution and homogeneity in the breast radiation treatment.

## Background

Breast cancer is one of the most spread cancer diseases, treated with different modalities. Adjuvant radiotherapy, after surgery, has been proven to increase the breast cancer specific survival [1]. However, the radiation treatment might increase the toxicity, cutaneous, cardiac and pulmonary, reducing the quality of life of the patients [2]. In 2002, after the introduction of the intensity modulated technique in breast cancer radiotherapy, Vicini et al. [3] evaluated the possible predicting factors for developing acute skin toxicity. Significant correlation (*p* = 0.005) in univariate and multivariate analysis was reported with dose homogeneity, in particular with the breast volume receiving 105 and 110% of the prescription dose (45 Gy delivered in 1.8 Gy/fraction in their work). The fractionation schemes have been changed in the last years, and hypofractionation is today widely used, with or without a simultaneous integrated boost. Such shorter schedules, mostly in 3 weeks, do not increase the toxicity relative to the previous conventional schedule on 5 weeks [4–7]. However, the statistical significance of the Vicini et al. data, although based on only 95 patients, suggested the importance of keeping the dose homogeneity in the breast as good as possible. Similarly, in 2015, Mak et al. [8] in a study on 280 patients reported that the breast tissue treated to more than 105 and 110% of the prescribed doses were found to be predictors of long term breast pain on univariate analysis, with the V_110%_ remaining significant also in a multivariate analysis with an odds ratio 1.01 per cm^3^, *p* = 0.007.

With the clinical implementation of the most advanced dose calculation algorithms, namely type ‘c’ [9] as Monte Carlo, the specific tissue anatomy in terms of its chemical composition can be properly taken into account to better estimate the physical dose distribution (and ultimately the dose homogeneity in the target). In particular, for breast cancer treatment, it is known that the mammary gland consists of lobules of connective tissue, separated by fat tissue, with the glandular fraction being assumed of about 40% of the whole breast. The female whole breast composition, including both glandular and fat fractions, according to the ICRP Publication 89 [10], presents lower carbon and higher oxygen fraction than fat. This might be consistent with the association of the lobular fraction to muscle tissue, having lower carbon and higher oxygen component than adipose tissue. The breast tissue composition in the two different fractions of lobular and fat compartments would in principle lead to different energy depositions (and dose) that could be better managed by dose calculation processes able to distinguish among different elemental composition of tissues, like Monte Carlo simulations, or algorithms as Acuros [11].

Aim of the present work is to estimate the dose variations generated by the difference in tissue chemical composition and not coming from the optimization process, which could compensate for dose differences when attempting to deliver homogeneous dose in the breast target (both lobular and fat fractions). Monte Carlo simulations were used herein, as well as Acuros as a clinically implemented dose calculation algorithm.

## Methods

### Treatment plan calculations

Five left breast cancer patients were selected from the institutional database. They were considered as a representative sample of the clinical practice. CT datasets were acquired in the supine position with 2 mm slice thickness, adjacent. Clinical target volume (CTV) was contoured on the CT dataset to encompass the whole mammary gland, and cropped 4 mm inside the skin. Additional structures were delineated: CTV_lob and CTV_fat, being the lobular and fat CTV volumes, respectively. These two last structures were contoured using a CT ranger, discriminating the two tissues with the HU = − 59 (CTV_fat where HU < − 59, CTV_lob where HU ≥ − 59, HU: Hounsfield Units). The ratio between the lobular and the fat volumes within the CTV was 0.21 ± 0.13 (range 0.11–0.40).

All the patients were planned with volumetric modulated arc therapy technique (VMAT), in its RapidArc form, on a 6 MV beam from a Varian TrueBeam linac equipped with a multileaf collimator Millennium-120 (Varian Medical Systems, Palo Alto, CA, USA). The arc geometry was of two partial arcs, with the gantry spanning from ~ 300 to ~ 170°, the collimator was of ~ ± 15°, set according to the breast shape and patient anatomy. Total dose prescription was 40.5 Gy in 15 fractions as mean CTV dose.

All the plans were generated with the Varian Eclipse treatment planning system, optimized with the Photon Optimizer (PO) algorithm (version 13.6) and calculated with Acuros XB (version 13.6). The same dose calculation algorithm was used to compute the dose distribution at least once during the plan optimization process (intermediate dose), to improve the optimization result according to an accurate dose estimation, in particular regarding the target dose homogeneity.

### Monte Carlo simulations

Patient CTs, structures and plans were exported in DICOM format from Eclipse and imported in PRIMO (version 0.3.1). PRIMO is a free computer software (http://www.primoproject.net) that simulates clinical linacs and estimates absorbed dose distributions in patient CT datasets (as well as in water phantoms) [12]. It combines a graphical user interface and a computation engine based on the Monte Carlo code PENELOPE [13–15]. A program for fast Monte Carlo simulation of coupled electron and photon transport, DPM, is also integrated [16], and used in the current work. The linac head was simulated by using the phase-space files made available by the linac vendor (Varian Medical Systems) for research purposes. Those phase-spaces were simulated into a Geant4 Monte Carlo environment and distributed according to the IAEA format [17]. In the current work, a phase-space for TrueBeam linac, 6 MV flattened beam quality, of 49.5e + 09 histories was used. Inside the patient, the transport parameters (to balance the trade-off between speed and accuracy) are predefined for DPM simulations as 50 and 200 keV cut-off energies for photons (bremsstrahlung) and electrons (collision), respectively. A variance reduction technique (splitting in CT with a factor 100) was used to reduce the computing time, that otherwise would be unacceptable if a direct approach was used. With this method, the average statistical uncertainty of all CT voxels accumulating more than 50% of the maximum absorbed dose, and reported by PRIMO at two standard deviations, was around 1% (range over all the simulations 0.99–1.08%).

### Tissue density and HU management

The same curve to convert HU to mass density was used in PRIMO and Acuros based systems. The material assignment based on the CT number was set in PRIMO as similar as possible to the Acuros setting in Eclipse. Full compatibility of the two assignments is not viable, since Acuros assigns adjacent materials in a smooth way, allowing an overlapping HU range, where the previous and next materials are linearly combined from one to the other. The used materials are summarized in Table 1.

**Table 1:** – HU and mass density ranges used in PRIMO and Acuros computations

	PRIMO HU range	PRIMO mass density range (g/cm^3^)	Acuros HU range	Acuros mass density range (g/cm^3^)
Air	− 1000, − 957	0, 0.0204	−1000, − 957	0, 0.0204
Lung	−957, − 400	0.0204, 0.594	− 967, − 374	0.0104, 0.624
Adipose	− 400, − 59	0.594, 0.969	− 434, 1	0.551, 1.001
Muscle	− 59, 88	0.969, 1.075	−59, 117	0.969, 1.093
Cartilage	88, 298	1.075, 1.199	57, 971	1.056, 1.600
Cortical bone	298, 2832	1.199, 2.833	128, 2832	1.100, 2.830

**Fig. 1:**
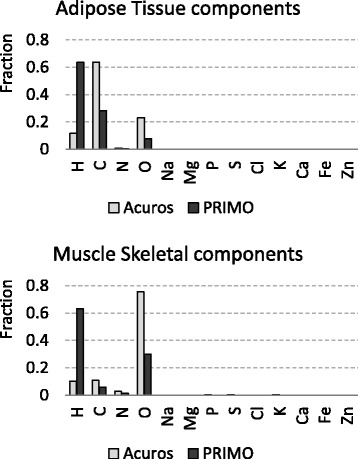
Default component fractions for Acuros and PRIMO. The Acuros values have been used in the current work

The specific chemical compositions as configured in the two systems, PRIMO and Acuros, are not identical in their defaults, being the hydrogen fraction in PRIMO higher than the corresponding fraction set for Acuros for most of the human tissues. To exclude a systematic error that could arise from this difference, the contribution of the various elements was modified in PRIMO for adipose and muscle tissues, to be more compatible with the Acuros materials. Figure 1 shows the elemental compositions of adipose and muscle tissues according to the PRIMO and Acuros defaults. The Acuros values were hence used in this work.

One of the patients of this study was simulated with the two chemical compositions for adipose and muscle tissues, according to the PRIMO and Acuros defaults. With the PRIMO defaults, the dose to muscle and adipose tissues were estimated higher than using Acuros defaults by about 0.12% and 0.03, respectively. Those differences, although considered negligible, were excluded from the computation by changing the PRIMO tissue composition material defaults.

### Patient doses with Monte Carlo simulations

For each of the five cases, three different Monte Carlo simulations were computed in PRIMO, assigning different materials to the muscle and adipose HU ranges, while keeping the original density:

- AdiMus: as standard, muscle and adipose tissues were assigned to the muscle and adipose HU ranges, respectively;

- Adi: the adipose tissue material was assigned to the HU including both adipose and muscle ranges;

- Mus: the muscle tissue material was assigned to the HU including both adipose and muscle ranges.

Mean doses to CTV, CTV_lob and CTV_fat were computed for all the simulations.

The dose difference generated by the chemical composition of the specific tissue, lobular or fat, was estimated by the difference of the mean doses of the CTV_lob between Adi and AdiMus simulations, and of the difference of the mean doses of the CTV_fat between Mus and AdiMus simulations. Those values give the possible dose estimation error when a different material chemical composition (adipose for lobular tissue, or muscle for fat tissue) is used for calculations, while the surrounding tissue dose is computed with the correct tissue assignment. Calculations were based on the mean dose of the whole structure. Uncertainties were reported at two standard deviations for all the voxels in each specific structure.

To include also the positional dose difference, the 3D gamma evaluation available in PRIMO software was analysed. The gamma index [18] was evaluated between AdiMus simulation (the best approximation of the true patient), and Adi or Mus simulations for CTV_lob and CTV_fat, respectively (i.e. assigning the “erroneous” material to the two portions, respectively). For the gamma criteria, the distance to agreement (DTA) was set to 2.5 mm, equal to the simulation grid, as well as to half of this value, 1.25 mm; the delta dose was varied from 0.5 to 3.0% of the maximum dose. No threshold dose value was limiting the evaluation, that was performed only inside the target (close to the prescription dose level). However, the analysis was restricted to the points with reference dose having uncertainty below 70%.

For one patient, two additional simulations were run, assigning to the HU range of the CTV the cartilage and the cortical bone tissues, keeping the original density. This would emphasize the importance of properly assigning the correct tissue (elemental composition) to the HU ranges.

### Comparison with Acuros calculations

Comparison of the PRIMO computed results was performed with Acuros calculations, as implemented in Eclipse (version 13.6). Acuros explicitly solve the Linear Boltzmann Transport Equation, while Monte Carlo methods (as PENELOPE in PRIMO) generate a stochastic solution by simulating a large finite number of particles. In principle, the two methods should lead to the same solution. However, non-negligible approximations are used in the radiotherapy planning practice. One of the most crucial is the material composition and assignment to pre-defined HU ranges, which is not modifiable in Acuros. This reason prevented the calculations in settings similar to the above-described Monte Carlo simulations (AdiMus, Adi, Mus). Nonetheless, to evaluate the dose difference generated by the elemental composition of tissues estimated by Acuros, dose calculations were performed also with AAA (Anisotropic Analytical Algorithm) implemented in Eclipse. The two algorithms used the same machine configuration data, and are based on the same concepts of the beam source model [19]. AAA does not take into account the specific tissue composition, and inhomogeneities are managed by rescaling the density according to HU, with no differentiation in the energy deposition for different materials (no medium differentiation). The differences arose in Acuros due to the chemical composition of the tissues were evaluated through the differences of the mean doses in CTV_lob and CTV_fat for Acuros and AAA calculations, once the two plans were renormalized to the same mean dose to CTV. This is clearly a very crude approximation to isolate the medium composition effect on the calculated dose.

## Results

### HU in lobular and fat breast portions

The analysed patients presented a mean HU of − 14 ± 10 and − 103 ± 3 in the lobular and fat portions of CTV, respectively. The Standard Deviations of the HU distributions inside CTV_lob and CTV_fat were 26 ± 2, and 21 ± 9, respectively. To notice is the quite stable HU values in the lobular and fat portions of breast among patients.

**Fig. 2:**
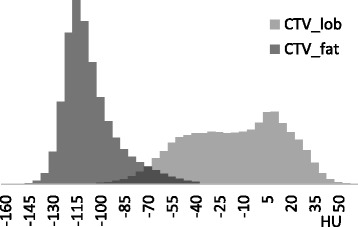
Average histograms on all the patients of the HU distributions of CTV_lob and CTV_fat

In Fig. 2 the average (over the analysed patients) HU histograms is presented, where the two peaks are well separated, although an overlap is present, due most probably to the structure contours inaccuracy (the CTV_lob was defined as the CTV voxels with HU larger than − 59).

### Monte Carlo simulations

**Fig. 3:**
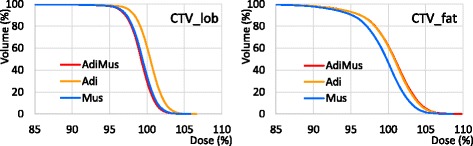
DVHs of lobular (left) and fat (right) portion of the breast from AdiMus, Adi and Mus Monte Carlo simulations

A cumulative dose-volume histogram example of one of the selected patients is presented in Fig. 3. Here, the CTV, CTV_lob and CTV_fat were presented for AdiMus, Adi, and Mus simulations. As expected, the AdiMus and Adi simulations estimated the same dose distributions in CTV_fat, while in CTV_lob this happens for AdiMus and Mus simulations.

Table 2 reports the percentage dose differences between the mean dose of the specific CTV portions of the test simulation, and the CTV mean dose from AdiMus simulations. The AdiMus CTV mean dose can be considered the standard condition for planning and dose prescription. The reported errors are the average statistical uncertainties in each specific structure, at 2 standard deviations, propagated for all the patients.

**Table 2:** - Percentage dose differences between the mean dose of the specific CTV portions of the test simulation and the CTV mean dose from AdiMus simulation

	AdiMus	Adi	Mus
CTV	0.00 ± 0.46%^a^	+ 0.20 ± 0.46%	− 0.86 ± 0.46%
CTV_lob	−0.61 ± 0.92%	+ 0.64 ± 0.92%	− 0.40 ± 0.92%
CTV_fat	+ 0.17 ± 0.49%	+ 0.09 ± 0.49%	−0.97 ± 0.49%

^a^dose prescription

The possible dose overestimation in the lobular breast region, relative to the prescribed dose, when adipose tissue is there assigned, is of 1.25 ± 0.45% (considering the difference of the mean doses from AdiMus and Adi simulations in the lobular fraction). Conversely, the possible dose underestimation in the fat region of the breast if muscle tissue is assigned is of 1.14 ± 0.51% (the differences of the mean doses from AdiMus and Mus simulations in the fat fraction). In the case of cartilage and bone assignments, a dose underestimation was evaluated of 0.6% and 2.8, respectively in the lobular fraction, and of 1.8% and 4.1 in the fat fraction.

All those differences are generated by the lone difference in elemental composition of the tissues, since the specific density of each voxel is allocated from the HU value.

**Fig. 4:**
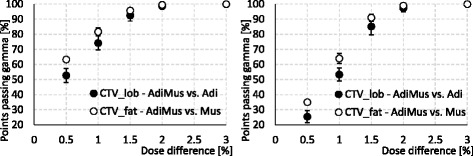
Gamma index analysis. Left: DTA = 2.5 mm; Right DTA = 1.25 mm. Error bars refer to the standard deviation among all the patients

The gamma evaluation analysis was summarized in Fig. 4, where the percentage of points fulfilling the criteria is shown for CTV_lob and CTV_fat comparing AdiMus vs. Adi and AdiMus vs. Mus simulations, respectively. From those graphs, a large amount of the structure volume is shown not to fulfil the criteria below a dose difference compatible with the difference estimated just above, between 1 and 1.5%.

The computed gamma evaluation presented an agreement for DTA = 2.5 mm and delta dose of 0.5% exceeding the 90–95% of the CTV_lob and CTV_fat volumes for AdiMus vs. Mus and AdiMus vs. Adi comparisons, respectively (that is between the simulations with muscle in the CTV_lob, and adipose in the CTV_fat, not shown in Fig. 4). This is consistent with the average uncertainty of the simulations, around 1% at two standard deviations.

### Acuros calculations

Concerning the clinical use of tissue differentiation in Acuros, the results showed a dose overestimation of the AAA (where no chemical composition is taken into account) in the lobular portion of breast of 0.98 ± 0.06%, and an underestimation of 0.21 ± 0.14% in the fat portion. Interesting to note is a better homogeneity between doses in the lobular and fat regions of the CTV found for the Acuros calculated plans, while the AAA recalculation presented an overdose to the lobular region of about 1%. The reason of an increased homogeneity in the Acuros calculated plan resides in the optimization process, which used Acuros calculation as intermediate dose to refine the optimization and improve the target dose homogeneity. If the optimization process uses a less accurate dose calculation algorithm for intermediate dose estimation (AAA), in these specific cases of breast planning, the lobular portion of then breast will be underdosed by 1%.

## Discussion

In this work, we analysed the dosimetric aspects of the whole breast irradiation arising from the special anatomy of the mammary gland, composed by two different tissues, the lobular and the fat connective tissue.

From the Monte Carlo data, there is a dose difference of more than 1% coming only from the chemical composition of the two different components. Such a difference most probably is not clinically significant, and is well within the accuracy required by the dose calculation systems. However, this systematic effect might produce an underdosage of such an amount of dose to the lobular fraction of the breast that is indeed the core of the mammary gland.

The works of Vicini et al. [3] and the more recent of Mak et al. [8] reported a significant correlation of the radiation effects, in terms of acute skin toxicity and long-term breast pain, to the breast volume receiving more than 105% or 110% of the prescription dose, whichever the dose fraction size. This correlation points to the need of delivering homogeneous dose in the breast, and in this frame a difference of 1–1.5% in the dose homogeneity could be of interest. However, the dose distributions calculated in the mentioned studies were affected by some systematic error due to the lack of knowledge in the tissue composition and related energy deposition, since none of those studies used so advanced calculation algorithms. A more accurate estimation of the dose distribution in the breast compartments could help the understanding of the correlation between toxicity and dose homogeneity.

The investigation of the dose effect of different breast compartments was already reported in 2011 [20], where dose calculations with Acuros showed this distinction with respect to AAA calculations, of about 1.6%, but more in a dose calculation algorithm comparison frame.

In this study, the plans were optimized with an inverse planning process, using intermediate dose calculations performed with the Acuros algorithm. This allowed a better homogeneity of the dose distribution inside the whole breast according to the same dose calculation algorithm. Being Acuros calculations more accurate than AAA in the inhomogeneity management, also thanks to the medium composition inclusion, the use of advanced calculations leads to more refined knowledge of the dose distribution, possibly improving the radiation treatment by modulating the dose according to the clinical effects on toxicity or outcome.

In the current work, we started from a pure Monte Carlo simulation, which is generally considered as the gold standard for the dose estimation. However, true Monte Carlo calculations are today not easily available in the clinical routine practice, due to the excessive long calculation time.

A problem that cannot be solved even with the Monte Carlo simulations refers to the approximation of the chemical composition and relative fractions of the different atomic components of human tissues. The human body is considered as composed by only six different media: air, lung, adipose, muscle, cartilage and bone, assuming that the tissue presenting HU in a certain range (from a CT dataset, that is a result of absorption) has exactly a defined proportion of some chemical components, as published for example in the ICRP Publication 89 [10]. This approximation is obviously not fully reflecting the real anatomy, and as a consequence, the dose estimation is affected by this approximation, even using the gold standard. The attempt to mitigate this issue was implemented in Acuros, using overlapping HU ranges between two adjacent tissues. On one side, this feature prevents the pure dose calculation comparison between full Monte Carlo and Acuros. On the other side, probably it better reflects the small differences in the human tissues, although keeping all the approximations and uncertainties. In the specific case of breast, the ICRP Publication 89 reported about the carbon and oxygen fraction difference between breast tissue (as a whole) and fat tissue, suggesting a trend to be more similar to the muscle tissue. However, the lobular fraction belongs to muscle medium in the HU ranges used for calculations, while it is not exactly muscle, and its specific chemical composition might be different.

These considerations on the human tissue compositions bring to one of the limitations of the current work. We analysed only the small variations in the breast tissue and their dosimetric consequences, i.e. the interface between adipose and muscle densities and compositions. What would be important to evaluate and estimate is the accuracy in calculation, or maybe the understanding the human tissues composition, in the other, more complex interfaces: air to lung, and cartilage to bone. For those two couples of tissues the distinction is much more complex, and more detailed studies in the specific anatomies would be advisable.

## Conclusion

A dose deposition difference in the lobular and connective fat fractions of the breast tissue is estimated with Monte Carlo simulations and Acuros calculations. Although not clinically significant, such a difference lead to an improved knowledge of the possible dose distribution and homogeneity in the breast radiation treatment.

## Abbreviations

AAA: anisotropic analytical algorithm
Adi: simulation with adipose assignment in both adipose and muscle CT number ranges
AdiMus: simulation with adipose and muscle assignments in adipose and muscle CT number ranges
CT: computed tomography
CTV: clinical target volume
CTV_fat: connective fat fraction of CTV
CTV_lob: lobular fraction of CTV
DTA: distance to agreement
HU: Hounsfield Unit
Mus: simulation with muscle assignment in both adipose and muscle CT number ranges
VMAT: volumetric modulated arc therapy
